# Interrogating Transcriptional Regulatory Sequences in Tol2-Mediated Xenopus Transgenics

**DOI:** 10.1371/journal.pone.0068548

**Published:** 2013-07-16

**Authors:** Gabriela G. Loots, Anne Bergmann, Nicholas R. Hum, Catherine E. Oldenburg, Andrea E. Wills, Na Hu, Ivan Ovcharenko, Richard M. Harland

**Affiliations:** 1 Biology and Biotechnology Division, Lawrence Livermore National Laboratory, Livermore, California, United States of America; 2 School of Natural Sciences, University of California Merced, Merced, California, United States of America; 3 Computational Biology Branch, National Center for Biotechnology Information, National Library of Medicine, National Institutes of Health, Bethesda, Maryland, United States of America; 4 Department of Molecular and Cell Biology, University of California, Berkeley, California, United States of America; Radboud University Nijmegen, The Netherlands

## Abstract

Identifying gene regulatory elements and their target genes in vertebrates remains a significant challenge. It is now recognized that transcriptional regulatory sequences are critical in orchestrating dynamic controls of tissue-specific gene expression during vertebrate development and in adult tissues, and that these elements can be positioned at great distances in relation to the promoters of the genes they control. While significant progress has been made in mapping DNA binding regions by combining chromatin immunoprecipitation and next generation sequencing, functional validation remains a limiting step in improving our ability to correlate *in silico* predictions with biological function. We recently developed a computational method that synergistically combines genome-wide gene-expression profiling, vertebrate genome comparisons, and transcription factor binding-site analysis to predict tissue-specific enhancers in the human genome. We applied this method to 270 genes highly expressed in skeletal muscle and predicted 190 putative *cis*-regulatory modules. Furthermore, we optimized Tol2 transgenic constructs in *Xenopus laevis* to interrogate 20 of these elements for their ability to function as skeletal muscle-specific transcriptional enhancers during embryonic development. We found 45% of these elements expressed only in the fast muscle fibers that are oriented in highly organized chevrons in the *Xenopus laevis* tadpole. Transcription factor binding site analysis identified >2 Mef2/MyoD sites within ∼200 bp regions in 6 of the validated enhancers, and systematic mutagenesis of these sites revealed that they are critical for the enhancer function. The data described herein introduces a new reporter system suitable for interrogating tissue-specific *cis*-regulatory elements which allows monitoring of enhancer activity in real time, throughout early stages of embryonic development, in *Xenopus*.

## Introduction

Many vertebrate genes exhibit highly intricate temporal and spatial expression patterns that span multiple stages of embryonic development, and specific embryonic or adult cell types. Several also respond to environmental and metabolic stimuli [1], [2]. While automated global gene expression methods, such as microarrays or tissue arrays have greatly expanded our view of the ‘transcriptome’ or where and when genes are expressed, we have yet to improve our ability to determine the transcriptional regulatory DNA sequences that are responsible for turning genes on and off, therefore understanding the regulatory mechanisms that drive expression of genes during development or in specific tissues remains one of the central problems in genome biology.

While transcription is mediated in part by promoters and promoter proximal elements that coordinate the site of initiation and contribute to the levels of transcription, distal *cis*-regulatory elements such as enhancers, insulators, locus control regions, and silencing elements control the stage and region-specificity of expression. These sequences are frequently positioned at great distances from the promoters of the genes they control. In addition, recent evidence suggests that exons can also function as transcriptional regulatory sequences of near-by genes [3]. Since regulatory elements can be present anywhere in the vicinity or within an open reading frame, and their sequence signatures are currently indistinguishable from non-functional DNA, only modest progress has been made in identifying distal enhancers in a high throughput manner, leaving most of the noncoding regions of mammalian genomes unannotated. Currently, the majority of the non-repetitive, noncoding genome of any sequenced vertebrate organism is of unknown function, through this ‘junk’ DNA may comprise a large fraction of the genome (estimated to be ∼50% of the genome in humans). However, increasing evidence suggests that a significant proportion of the noncoding sequences, possibly up to 80% (encode papers) contain regulatory elements responsible for the elaborate expression programs in the diverse cell types of the vertebrate body.

While comparative sequence analysis has emerged as an efficient tool for prioritizing noncoding sequences likely to function as transcriptional regulatory elements *in vivo*, conservation alone has failed to advance our ability to predict the tissue-specificity of a regulatory element, *de novo*. For example, while searching for a bone enhancer in a 52 kb human deletion known to caused Van Buchem disease and harboring 17 human to mouse conserved noncoding sequences (>100 bp; >70% sequence identity, only one of the seven tested sequences displayed enhancer activity, in the UMR-106 osteosarcoma cell line [4]. Furthermore, this element had to be validated *in vivo*, in transgenic mice to confirm its tissue specificity in the mouse skeleton [5].

During the last decade, we and others have developed computational and experimental methods that would help address several unresolved questions: Where are distal *cis*-regulatory sequences located in vertebrate genomes? Are they likely to control single or multiple transcripts? Do they activate expression in single or multiple cell types? Is there a ‘code’ associated with cell type specific distal enhancers? The ENCODE project has already initiated work that addresses some of these questions and recent valuable insights have been obtained that describe new chromatin patterns at transcription factor binding sites [6], chromatin modification patterns around promoters that may influence tissue specificity [7] as well as DNA methylation profiles linked to specific regulatory elements [8]. While all these insightful approaches have greatly expanded our view of the human genome’s regulatory landscape, two important aspects have not been addressed by any ENCODE project, mainly can we predict and annotate tissue specific enhancers, *de novo*? Most importantly it remains to be determined how the data derived from cell lines translates to the full organismal level. Since our existing knowledge of distal regulatory elements is very limited, for significant progress to occur, we primarily need to improve our available methods for testing and characterizing transcriptional regulatory elements, *in vivo,* to expand our repertoire of ‘validated’ tissue specific enhancers, which could subsequently be used to further our understanding of the sequence signatures or ‘regulatory code’ of vertebrate genome.

Using a transposon mediated transgenesis method originally developed in Zebrafish [9] and more recently optimized in both *Xenopus laevis* and *tropicalis* [9]–[11] here we describe a general purpose transgenesis construct that can be used in transient transgenic frogs to interrogate potential transcriptional regulatory sequences for their ability to function as tissue-specific transcriptional enhancers during embryonic development. This procedure is based on Tol2 transposable elements isolated from the fish *Orzyias latipes* (Medaka), where a circular plasmid containing the transgenic construct flanked by Tol2 arms is injected into fertilized eggs along with the Tol2 transposase RNA. In building a suitable reporter system amenable to high throughput validation of distal *cis*-regulatory elements we aimed to construct a vector that would incorporate: (1) a minimal promoter that is either ‘off’ in the absence of a strong transcriptional enhancer, or is ‘on’ in a highly restrictive manner, for example it only expresses reproducibly in a subset of cell types and can be used as a positive control for transgensis; and (2) a fluorescent reporter gene that is bright, stable and folds relatively quickly to allow us to monitor enhancer activity in real time during early stages of development.

We also wanted our system to yield high numbers of transgenic embryos that express robust levels of the transgenic protein in a reproducible pattern to allow us to reliably analyze first generation transgenic embryos (G0) without having to pass the transgenes through the germline. Here we report that gamma-crystallin (*γ-cry*) represents an ideal minimal promoter for interrogating transcriptional *cis*-regulatory elements in Tol2-mediated transgenic *Xenopus laevis* embryos. The expression of the red fluorescent protein mKate2 in the lens beyond embryonic stage 35 allows us to assess transgenic efficiency in the absence of enhancer activity. Using three known tissue specific enhancers (one kidney and two skeletal muscle enhancers) we optimized transgenic efficiency to obtain up to 93% transgenic embryos among the surviving tadpoles. Furthermore, we proceeded to correlate tissue specific expression of candidate muscle enhancer elements predicted using a computational methods previously described (Pennacchio et al. 2007), and found 55% (11/20) of the tested elements to exhibit enhancer activity, where 45% (9/11) specifically drove expression in the predicted tissue. This simple and efficient technique represents a powerful new tool for high-throughput transgenic analysis of candidate *cis*-regulatory elements. Germ line transmission of Tol2 *X. tropicalis* transgenic embryos generated using validated enhancer elements will facilitate the generation of tissue-specific reporter transgenic lines that can be used in future genetic studies in the diploid *X. tropicalis*.

One great advantage of using Xenopus over the gold standard set by the mouse is the ability to view gene expression during early embryonic development in real time. Because of *in utero* development, transient transgenic expression in mice can only be characterized at specific time points during development. To be able to capture the transgene expression, one needs to know when and where the controlled transcript is expressed in order to predict the developmental window a putative *cis*-regulatory element is likely to function. Since gene expression for most genes is highly dynamic, in the absence of such detailed transcriptional information, *cis*-regulatory predictions focusing on characterization at a single time point are likely to miss a plethora of elements that act earlier or later in development. Pennacchio *et al*. examined over 2000 conserved noncoding elements in transgenic mice at E11.5 and found ∼30% of these elements to generate reproducible expression patterns [12], some of which may have been missed due to the selected time point. The transgenic approach describe here represents an alternative method for examining the regulatory landscape in vertebrates and is likely to facilitate the discovery of novel DNA sequences with transcriptional regulatory potential.

## Results

### Tol2 Transgenesis Provides High Survival and High Transgenic Efficiency in G0 Embryos

**Figure 1:**
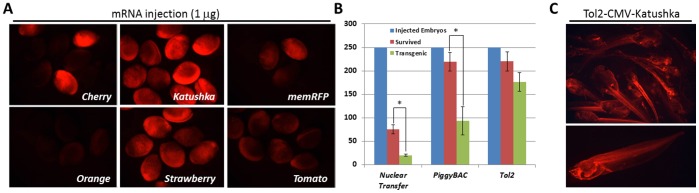
Comparing fluorescent reporter expression and transgenic efficiency in transgenic embryos generated by nuclear transfer, PiggyBAC or Tol2 transgenesis. Six red fluorescent proteins were examined for bright expression in Xenopus oocytes (A). Using the brightest red fluorescent gene, Katushka, constructs with a ubiquitous promoter (CMV) were examined by three methods: nuclear transfer, PiggyBAC transposition and Tol2 transposition. 250 embryos were injected in four independent experiments using CMV-Katushka constructs, and the number of surviving and transgenic embryos were assessed (Table S1); significantly fewer transgenic embryos (*p*-value <0.005) were generated by nuclear transfer and PiggyBAC, where large numbers of the surviving Tol2 embryos were transgenic (B). Tol2 transgenics were found to give the brightest and most reliable expression in Xenopus (C).

Genetically encoded fluorescent proteins have been widely used as transgenic reporters and have been primarily optimized for mammalian *in vitro* and *in vivo* applications; among them green fluorescent protein (GFP) has been most widely used. One of the great challenges associated with GFP *in vivo* imaging in embryos involves minimizing the effects of background fluorescence, or autofluorescence. Autofluorescence is a fluorescent signal derived from substances other than the fluorophore of interest, and inherently animal tissues tend to display high autofluorescence levels in the visible wavelength range. While autofluorescence can be chemically quenched in fixed tissues, this becomes a challenge when imaging live tissues that have low transgenic expression levels. Recent reports have examined a family of far-red fluorescent proteins and have shown that both in tissue culture and in animals, these fluorescent proteins have high fluorescence resonance energy transfer efficiency (brighter); they also take advantage of the reduced light scattering associated with far-red illumination (allowing deep tissue imaging), and reduced excitation of yolk and other autofluorescent proteins, suggesting that they are well suited for real-time *in vivo* imaging [13]. We examined several far-red proteins for their ability to generate a strong signal in live embryos. We injected 1 µg of synthetic Cherry, Katushka, membrane RFP (mRFP), Orange, Strawberry and Tomato mRNAs into fertilized embryos and imaged them 20 hours post injection (Fig. 1A) (stages 18–20). We found Cherry and Katushka to have the highest fluorescence intensity, followed by Strawberry, Tomato and membrane RFP which were moderately bright and lastly Orange which had the weakest signal (Fig. 1A).

Using the ubiquitous human cytomegalovirus (CMV) and EF1A promoters driving Katushka red fluorescent protein we compared transgenic efficiency using two previously described transgenic methods: restriction enzyme mediated integration (REMI) and transposon mediated transgenesis. Based on previously reported transgenic success, we chose to examine two different transposon systems: PiggyBAC [14] and Tol2 [9], [10], [15] to determine which transgenic method yields the highest transgenic efficiency. We built plasmids that carried the CMV-Katushka or EF1A-Katushka cassettes flanked by PiggyBAC or Tol2 arms [9], [10], [14], [16]. These promoters have been widely used in a variety of vertebrate species and their activity has already been characterized by REMI and Sce-I mediated transgenesis in *X. laevis* and *X. tropicalis* [17] hence they provide a robust assay for assessing the efficacy of these methods. All experiments were performed on groups of 250 embryos per experiment (N = 4), and with equal amounts of DNA or DNA+RNA ratios. Nuclear transfer was carried out as previously described [18], [19]. For the transposon mediated transgenesis 200 pg of plasmid DNA was co-injected with 200 pg of freshly synthesized transposon mRNA at the 1-cell stage. After the injection, Katushka expression in the embryos was examined daily using an epifluorescence microscope up to stage 45, and scoring was carried out primarily between stages 35 and 40. For each experiment we tabulated the number of surviving tadpoles, the number of transgenic embryos, and the level of transgenic expression. We classified the transgenic expression intensity in three categories: ubiquitous (expression throughout the embryo), low mosaic (a small subset of the embryo) or high mosaic (spotty expression but covering more than 50% of the embryo).

Of the 250 injected embryos, we observed the lowest survival rate for the nuclear transfer method, averaging ∼30% survival (75/250). In contrast, both transposon mediated transgenic procedures had robust survival rates of up to ∼88% (220/250) (Fig. 1B; Sup. Table 1). Tol2 transgenesis outperformed the other two methods with transgenic rates as high as 90% compared to 34% for REMI, but expression intensity was variable and mosaic where 50% embryos had ubiquitous expression, 30% had high chimeric expression and 20% had low chimeric expression (Fig. 1C; Table S1). PiggyBAC generated the least reliable results in our hands. While the viability and transgenic rate were higher than REMI, the majority of the transgenic embryos had very low expression level (334/372) and the transgene was expressed at random in subsets of cells. With both PiggyBAC and Tol2, we also observed low level random transgenic expression in DNA injections without transposase RNA. Despite the high sensitivity of the nuclear transfer method, the high morbidity rate suggested that the Tol2 transgenic method has a higher potential for evolving into a reliable approach for validating and characterizing *cis*-regulatory elements in a transient transgenic assay. All subsequent transgenic experiments were carried out using Tol2-mediated transgenesis.

**Table 1:** Evaluating promoter and enhancer specificity to determine optimum transgenic construc configuration for enhancer characterization.

	*β-globin* (none)				*γ-cry* (eye)			
	Transgenic Efficiency	Eye/Skin	Muscle/Kidney	Other	Transgenic Efficiency	Eye/Skin	Muscle/Kidney	Other
Promoter Only	0/173	0	0	0	43/56 (76%)	100%	0	0
IRX3 KE	27/101 (27%)	0	56%	44%	219/282 (77%)	60%	40%	0
Dystophin ME	97/202 (48%)	0	98%	2%	76/179 (42%)	47%	53%	0
MyoD ME	93/133 (70%)	0	100%	0	169/181 (93%)	4%	96%	0
	***Irx3*** (eye)				***Krt8*** (skin)			
	Transgenic Efficiency	Eye/Skin	Muscle/Kidney	Other	Transgenic Efficiency	Eye/Skin	Muscle/Kidney	Other
Promoter Only	25/138 (18%)	60%	28%	22%	167/211 (79%)	80%	3%	17%
IRX3 KE	47/97 (48%)	30%	60%	10%	161/214 (75%)	60%	24%	16%
Dystophin ME	80/199 (40%)	2%	96%	2%	156/177 (88%)	25%	69%	6%
MyoD ME	87/173 (50%)	2%	98%	0	167/178 (93%)	9%	89%	2%
	***Hsp68*** (none)							
	Transgenic Efficiency	Eye/Skin	Muscle/Kidney	Other				
Promoter Only	86/110 (78%)	20%	56%	24%				
IRX3 KE	79/99 (79%)	21%	49%	30%				
Dystophin ME	162/182 (89%)	10%	75%	15%				
MyoD ME	205/240 (85%)	12%	78%	10%				

### Keratin 8 and γ-crystallin Promoters Drive Robust Enhancer-specific Expression in Xenopus

An ideal minimal promoter suitable for interrogating transcriptional enhancers would be either silent or ‘on’ in a highly specific subset of cells, yet would show consistent activation of tissue-specific expression at high levels when paired with an enhancer element. Several minimal promoters have been successfully used in mouse transient or stable transgenic lines, including *Hsp68* and *β-globin*. Among >1200 putative enhancers tested using the *Hsp68* promoter in mice, a significant fraction of the ∼600 elements with enhancer activity had neuronal specific patterns, suggesting that Hsp68 may be prone to non-specific activation of transgenes in the nervous system [12] (enhancer.lbl.gov). The *β-globin* promoter has been shown to be less susceptible to non-specific activation, however, we find this promoter to be highly specific but less sensitive and provides weaker expression level, in transgenic mice [5]. In zebrafish the keratin 8 (*Krt8*) promoter has been successfully used for both enhancer validation as well as for enhancer trapping [20], [21], and gamma-crystallin (*γ-cry*) has been frequently used in Xenopus transgenic assays as a positive control [22]. The *Krt8* promoter drives expression on the surface layer of stratified epithelial tissues primarily in the skin epidermis while the *γ-cry* promoter is specific for the lens.

**Figure 2:**
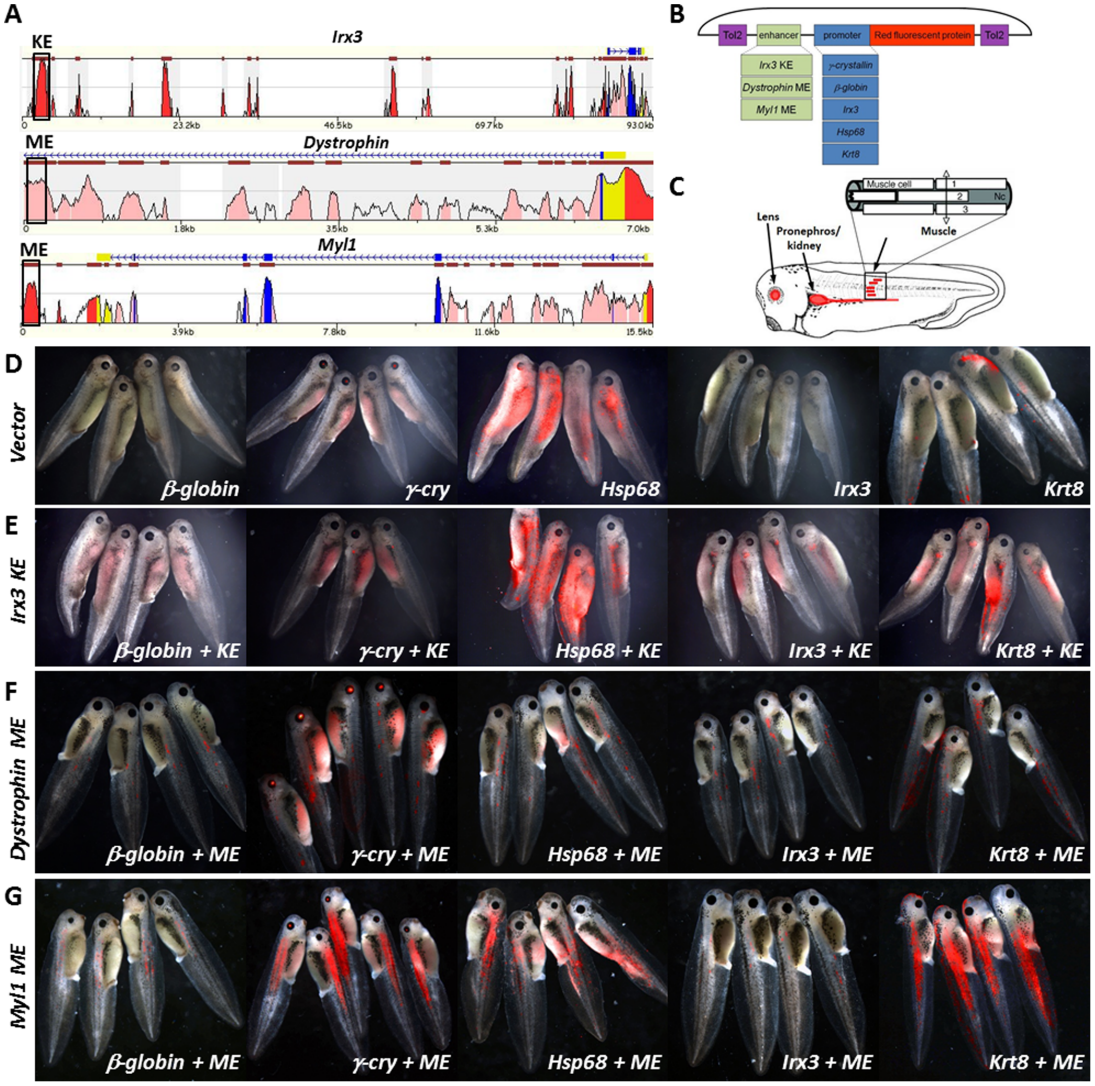
Characterizing minimal promoters for their ability to drive enhancer expression in a robust and reproducible manner. Three evolutionary conserved and previously characterized enhancers were examined as positive controls (A), in combination with 5 minimal promoters that have been previously employed in transgenic experiments in mice, zebrafish and frog (B). Frog embryos were examined for eye, kidney and muscle expression between stages 30–45 of embryonic development (C) in all enhancer-promoter combinations (D–G).

As initial test enhancers, we selected the *Irx3* kidney enhancer (KE) that has been previously characterized in *Xenopus* [23] along with two rodent-derived skeletal muscle enhancers (ME) [24], [25]. The kidney enhancer (KE) is 2 kilobases (kb) in length, is positioned ∼90 K upstream of the *Irx3* transcript and is highly conserved from man to fish (Fig. 2A). The dystrophin ME enhancer is 529 basepairs (bp) in length and positioned within the first intron of the dystrophin mouse gene. It was originally found to activate transcription in myogenic cells in both cell lines and transgenic mice [25]. The Myosin light chain 1/3 ME enhancer is 955 bp in length, is positioned downstream of the rat Myosin light chain 1/3 gene (*Myl1*) and has been validated in primary muscle cells derived from both mice and rats [24]. Both MEs are highly conserved from human to mouse (Fig. 2A), but not conserved in chicken, frog nor fish as evaluated by sequence alignments in their respective genomic loci. The KE and ME enhancers were cloned in front of the *β-globin*, *γ-cry*, *Hsp68*, *Irx3* and *Krt8* promoters in the T2 vector and were tested in *X. laevis* embryos (Fig. 2B). Transgenic expression was evaluated in live embryos between embryonic stages 30–45 monitoring enhancer- and promoter-specific target tissues (Fig. 2C). The *β-globin* and *Irx3* promoter only constructs gave very little or no signal, the *γ-cry* promoter showed high signal with 100% eye specificity, the *Krt8* promoter displayed varying degrees of low level skin expression, and the *Hsp68* construct exhibited high frequency, high intensity, random expression patterns (Fig. 2D; Table 1). In combination with KE, the *γ-cry* and *Irx3* promoters generated the most specific expression patterns (Fig. 2E), where *γ-cry* continued to show 100% target specificity for both promoter and/or enhancer expression, and the *Irx3* construct had ∼10% random non-specific expression. The *Krt8* had the brightest kidney expression but the relative frequency of transgenic embryos with kidney expression was low (24%) and a large number of transgenic embryos had high, random expression patterns (16%). The *Hsp68* construct continued to display the largest number of very bright, non-enhancer specific expression with >50% of transgenic embryos expressing in eye, skin, muscle or tissues other than kidney (Fig. 2E; Table 1). The *β-globin* promoter was the least responsive to the KE (27% transgenesis), and among the transgenics the expression level was very low and relatively non-specific (Fig. 2E; Table 1).

The dystrophin ME was a relatively weak enhancer, and only the *Krt8* promoter was robustly activated while all other promoters showed transgene expression in a few muscle cells (Fig. 2F). The *Myl1* ME was a much stronger enhancer, the *γ-cry* and *Krt8* promoters gave the highest rate of transgenic embryos (93%), high specificity (96% and 89%) and very bright expression, while the *β-globin* and *Irx3* had the lowest transgenic rates (70% and 50%) and very weak transgenic expression (Fig. 2G). While the strong ME enhancer displayed the lowest non-specific expression in all constructs, the *γ-cry* and *Krt8* consistently exhibited the strongest expression intensity, with *γ-cry* outperforming *Krt8* in tissue-specificity. *γ-cry* in combination with all 3 enhancers had 100% tissue-specificity and was shielded from position effect. Based on this assessment we determined that *γ-cry* may be the most suitable promoter for interrogating *cis*-regulatory elements in Tol2-transgenic *Xenopus* due to its high specificity of enhancer activation, built in positive control and lack of non-specific transgene activation, however it may miss the activity of elements with weak enhancer action, in which case *Krt8* may represent a better choice but may require the analysis of larger groups of embryos to determine enhancer specificity.

### Validating Computational Prediction for Muscle-Specific Enhancers

The genomic footprint of transcriptional regulation consists of modules of transcription factor binding sites (TFBS) residing within gene regulatory elements. Tissue-specific regulatory elements are thought to harbor similar TFBS modules characteristic of particular transcription factors (TFs) involved in gene regulatory programs specific to that tissue. From the computational perspective, the presence of similar TFBS combinations in a set of sequences necessitates the use of pattern search approaches capable of extracting the sets of TFBS that are characteristic to an input set of co-functional regulatory elements. In practice, large sets of tissue-specific regulatory elements are rarely available. Genome-wide gene expression experiments (gene expression arrays and RNA-Seq studies) usually retrieve sets of co-expressed genes, each with a locus full of many potential regulatory elements and only few that are active specifically in the tissue of interest. This creates a computational challenge when a set of putative regulatory elements should be extracted from a set of loci of co-expressed genes. Then, these sets of elements (one per locus) would be combined and compared to identify a small number of elements in each locus that share their composition of TFBS across the set of gene loci, arguing for the functional similarity within this set of elements that corresponds to the function of genes and is reflective of tissue-specific enhancer function of these elements.

We have previously developed a computational approach called Enhancer Identification or EI [26] that predicts tissue-specific enhancers and their characteristic TFBS modules from a set of genes co-expressed in a particular tissue. We applied EI to a set of 300 genes determined to be highly expressed in skeletal muscle according to the GNF Novartis Atlas of gene expression in mouse tissues [27]. This method detected a set of 190 putative skeletal muscle enhancers (SME) within the loci of these transcripts, with 56% of them residing distal to the nearest promoter region (Table S2). A follow up analysis of TFBS combinations shared by the predicted SMEs revealed a pronounced presence of MEF2, SRF, MyoD, Myogenin, and UBP1 (aka LBP1) TFBSs, with MEF2 sites being present in 40% of the predicted SMEs. While the first four of these TFs (including MEF2) have been shown to play key roles during muscle development (Li et al. 2005; Naya and Olson 1999), little is known about UBP1 except that it regulates extra-embryonic angiogenesis (Parekh et al. 2004).

**Figure 3:**
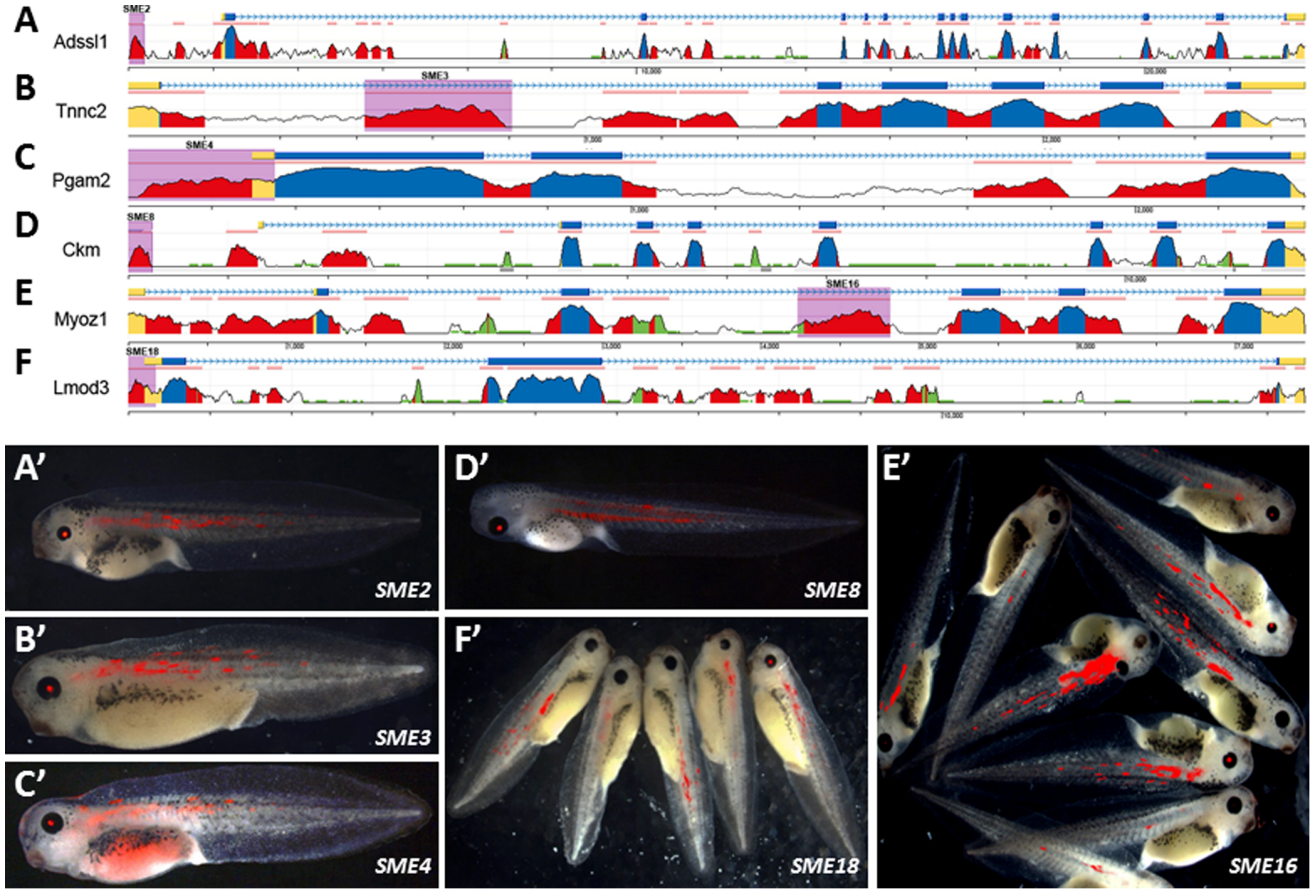
Validating computational predictions for muscle enhancers in *Xenopus* trangenics. Predicted SMEs corresponded to evolutionary conserved elements proximal or distal to genes known to function in skeletal muscle (A-F). Six SMEs consistently expressed in skeletal muscle, in addition to eye (A′–F′). Genomic regions are color coded as follows: exons (blue); UTRs (yellow); repeats (green); conserved noncoding sequences (red); predicted SMEs are shaded in purple.

The top 20 SME predictions were examined in frog enhancer assays, using both the *γ-cry* and *Krt8* Tol2 transgenic constructs described above. Eleven or 55% of these predicted elements showed tissue specific activity. Six elements displayed muscle-enhancer activity in combination with both the *γ-cry* and the *Krt8* promoter (Fig. 3), and 3 additional elements were weakly positive only with the *Krt8* promoter (Table 2; Fig. S1A–C). Two other elements had enhancer activity in tissues other than muscle, one displaying a neuronal (SME12) and the other a skin pattern (SME14; Table 2; Fig. S1D–E); only one of these elements was positive in combination with *Krt8* promoter; SME12 did not come up positive for hindbrain in combination with the *Krt8* promoter most likely because the skin expression masked the underlying hindbrain expression observed with *γ-cry*. Three of the 9 confirmed SMEs were intronic, four were positioned within 5′UTRs, one was intergenic and one was within a promoter region (Table 2; Fig. 3A–F). All genes associated with the SMEs confirmed in both the *γ-cry* and *Krt8* promoter constructs have been previously shown to function in skeletal muscle and included: adenylosuccinate synthase like 1 (*Adssl1*) [28], troponin C type 2 (*Tnnc2*) [29], phosphoglycerate mutase 2 (*Pgam2*) [30], muscle creatine kinase (*Ckm*) [31], myozenin 1 (*Myoz1*) [32] and Leiomodin 3 (*Lmod3*) [33]. Only one of the three elements confirmed with the *Krt8* promoter only was associated with a gene previously shown to function in skeletal muscle: integrin beta 1 binding protein 2 or melucin (*Itgb1bp2*) [34], while the other genes Solute carrier family 25-member 25 (*Slc25A25*) and fat storage inducing transmembrane protein 1 (*Fitm1*) have not yet been examined in skeletal muscle. All SMEs except SME8 correspond to novel muscle specific regulatory elements; SME8 overlaps with the 206-bp MCK enhancer that has been previously characterized in muscle cell lines and in transgenic mice [35].

**Table 2:** The top 20****SME computational predictions were examined in combination with the *γ-cry* and the *Krt8* promoter.

-cry-promoter constructs												
	Genomic	Adjacent		# Embryos		Transgenic Expression				% Tissue		Enhancer
ID	Element Location (mm9)	Context	Gene	Score	Injected	Survived	Total	Eye	Muscle	Other	Specificity	Function
SME1	chr2∶32302688–32303221	intron	Slc25a25	23.495	300	248	232	225	5	0	0	No
***SME2***	***chr12∶113856421–113856740***	***intergenic***	***Adssl1***	***18.945***	***380***	***212***	***194***	***63***	***130***	***6***	***67***	***Muscle***
***SME3***	***chr2∶164604395–164604716***	***intron***	***Tnnc2***	***13.504***	***610***	***357***	***305***	***227***	***77***	***9***	***25***	***Muscle***
***SME4***	***chr11∶5703692–5703983***	***UTR5***	***Pgam2***	***13.226***	***430***	***231***	***163***	***103***	***58***	***2***	***35***	***Muscle***
SME5	chr6∶14705247–14705528	UTR5	Ppp1r3a	11.861	260	198	148	140	8	0	0	No
SME6	chr8∶126408666–126408924	intergenic	Acta1	11.434	1080	353	220	205	3	12	0	No
SME7	chr5∶130347257–130347469	intron	Phkg1	10.212	170	120	93	93	0	0	0	No
***SME8***	***chr7∶19995139–19995384***	***promoter***	***Ckm***	***9.821***	***400***	***254***	***235***	***18***	***216***	***1***	***92***	***Muscle***
SME9	chr14∶27707593–27708097	UTR5	Asb14	9.493	200	125	97	97	0	0	0	No
SME10	chr14∶56194193–56194518	UTR5	Fitm1	8.859	300	155	97	89	8	0	0	No
SME11	chrX:98644054–98644511	UTR5	Itgb1bp2	8.103	330	253	146	139	7	0	0	No
SME12*	chr10∶17515705–17516036	UTR5	Txlnb	8.057	310	188	96	56	0	38	39	Hindbrain
SME13	chrX:154136273–154136451	promoter	Smpx	7.602	100	82	69	69	0	0	0	No
SME14*	chr3∶151927751–151928151	intron	Nexn	7.519	375	266	173	129	0	44	25	Skin
SME15	chr3∶102877718–102878018	UTR5	Ampd1	7.469	300	170	51	51	0	0	0	No
***SME16***	***chr14∶21470944–21471529***	***intron***	***Myoz1***	***7.175***	***670***	***343***	***213***	***123***	***125***	***25***	***58***	***Muscle***
SME17	chr1∶164569192–164569394	UTR5	Myoc	7.08	500	237	134	131	0	3	0	No
***SME18***	***chr6∶97202653–97202980***	***UTR5***	***Lmod3***	***6.786***	***300***	***250***	***157***	***99***	***58***	***1***	***37***	***Muscle***
SME19	chr6∶29381445–29381811	promoter	Flnc	6.756	400	140	104	101	2	3	0	No
SME20	chr9∶121686568–121686893	UTR5	Kbtbd5	6.461	300	156	97	95	0	2	0	No
**Krt8-promoter constructs**												
	**Genomic**	**Adjacent**		**# Embryos**		**Transgenic Expression**				**% Tissue**		**Enhancer**
**ID**	**Element Location (mm9)**	**Context**	**Gene**	**Score**	**Injected**	**Survived**	**Total**	**Eye**	**Muscle**	**Other**	**Specificity**	**Function**
***SME1***	***chr2∶32302688–32303221***	***intron***	***Slc25a25***	***23.495***	***770***	***445***	***312***	***197***	***100***	***15***	***32***	***Muscle***
***SME2***	***chr12∶113856421–113856740***	***intergenic***	***Adssl1***	***18.945***	***560***	***149***	***102***	***46***	***72***	***0***	***70***	***Muscle***
***SME3***	***chr2∶164604395–164604716***	***intron***	***Tnnc2***	***13.504***	***490***	***177***	***99***	***59***	***36***	***4***	***35***	***Muscle***
***SME4***	***chr11∶5703692–5703983***	***UTR5***	***Pgam2***	***13.226***	***430***	***231***	***163***	***103***	***58***	***2***	***35***	***Muscle***
SME5	chr6∶14705247–14705528	UTR5	Ppp1r3a	11.861	420	153	87	67	20	0	23	No
SME6	chr8∶126408666–126408924	intergenic	Acta1	11.434	275	202	147	147	0	2	0	No
SME7	chr5∶130347257–130347469	intron	Phkg1	10.212	420	225	135	99	15	21	11	No
***SME8***	***chr7∶19995139–19995384***	***promoter***	***Ckm***	***9.821***	***840***	***456***	***353***	***105***	***230***	***18***	***65***	***Muscle***
SME9	chr14∶27707593–27708097	UTR5	Asb14	9.493	420	139	104	76	21	7	20	No
***SME10***	***chr14∶56194193–56194518***	***UTR5***	***Fitm1***	***8.859***	***420***	***263***	***79***	***40***	***36***	***3***	***45***	***Muscle***
***SME11***	***chrX:98644054–98644511***	***UTR5***	***Itgb1bp2***	***8.103***	***420***	***188***	***161***	***76***	***73***	***12***	***45***	***Muscle***
SME12	chr10∶17515705–17516036	UTR5	Txlnb	8.057	280	184	149	146	3	0	2	No
SME13	chrX:154136273–154136451	promoter	Smpx	7.602	240	125	74	72	2	0	2.7	No
SME14*	chr3∶151927751–151928151	intron	Nexn	7.519	240	150	110	76	2	32	42	Skin
SME15	chr3∶102877718–102878018	UTR5	Ampd1	7.469	100	62	53	51	1	1	1.8	No
***SME16***	***chr14∶21470944–21471529***	***intron***	***Myoz1***	***7.175***	***100***	***38***	***28***	***16***	***11***	***1***	***39***	***Muscle***
SME17	chr1∶164569192–164569394	UTR5	Myoc	7.08	100	57	134	18	0	0	0	No
***SME18***	***chr6∶97202653–97202980***	***UTR5***	***Lmod3***	***6.786***	***160***	***44***	***34***	***15***	***19***	***0***	***56***	***Muscle***
SME19	chr6∶29381445–29381811	promoter	Flnc	6.756	100	40	104	23	0	0	2	No
SME20	chr9∶121686568–121686893	UTR5	Kbtbd5	6.461	100	52	37	35	0	1	0	No

* elements show enhancer activity in a tissue other than the predicted tissue.

### Mef2 and MyoD TFBS are Critical for SME8 and SME16 Function

**Figure 4:**
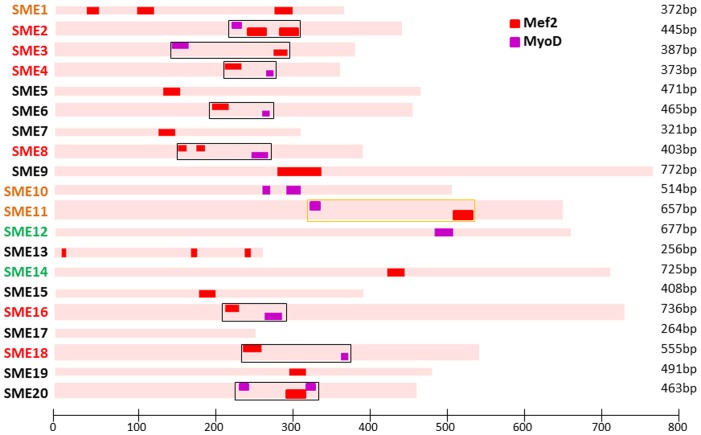
TFBS analysis. Top 20 predicted SMEs were examined for the presence of Mef2 and MyoD clusters. We found clusters of >2 Mef2/MyoD sites over regions >200 bp in all 6 SMEs shown to drive muscle expression in combination with *γ-cry* promoter (red) and 1/3 of the additional 3 enhancers that were found to drive muscle expression in combination with *krt8* promoter only (orange). Two of the negative elements also displayed this cluster (SME6/SME20), but none of the enhancers found to express in tissues other than muscle (green).

**Figure 5:**
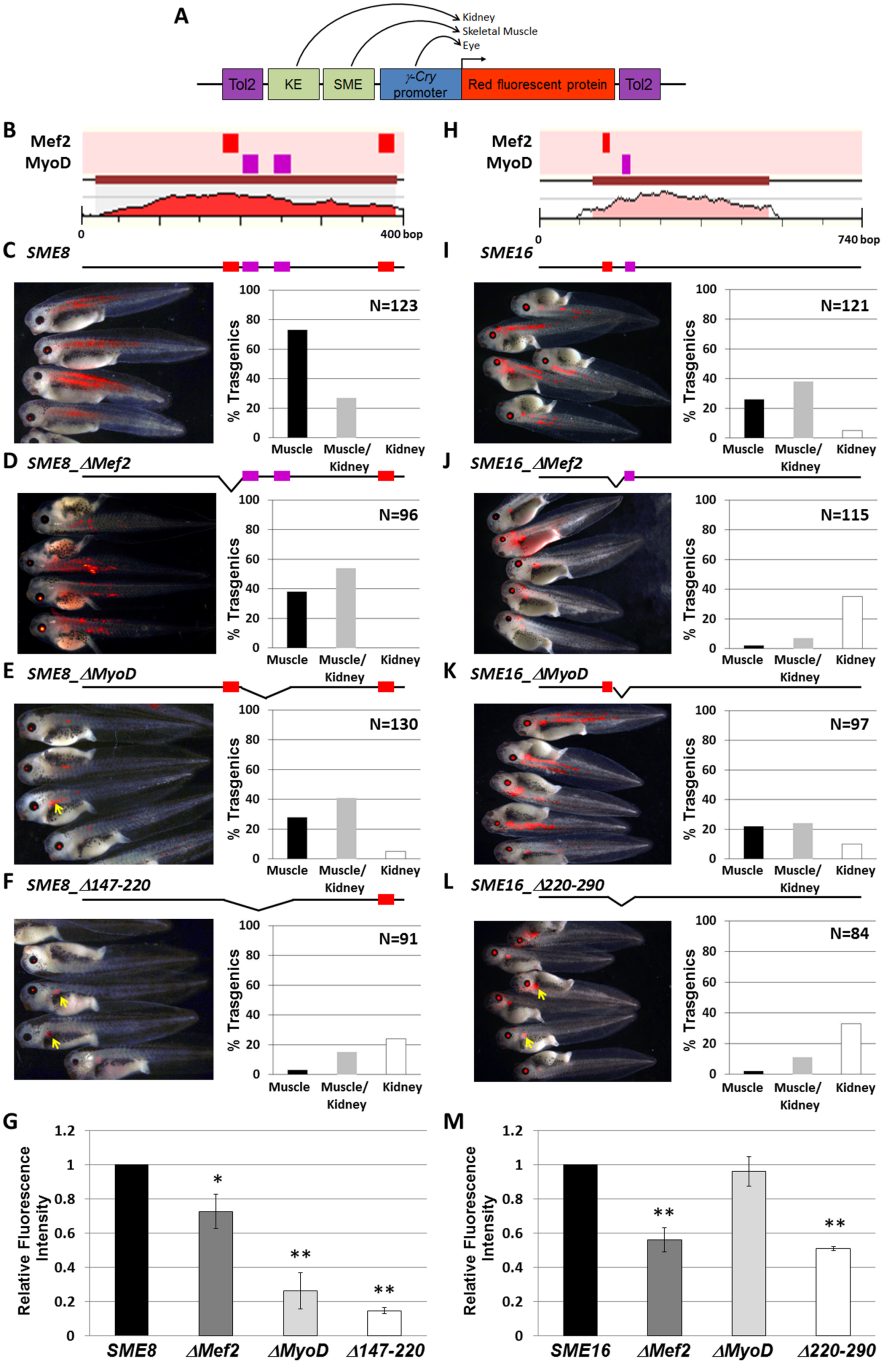
Mef2C/MyoD sites are essential for SME8/16 muscle specific expression. Tol2 constructs containing tandem kidney (KE) and muscle (SME) enhancers in front of *γ-cry* promoter (A) were systematically mutated to remove the predicted Mef2 and MyoD sites (B, H), and compared to the ‘wildtype’ construct in transgenic efficiency and tissue specificity (C, H), as well as expression intensity (G, M). Mutating either Mef2C or MyoD sites reduced the number of embryos expressing in muscle, as well as reduced the expression intensity (D-F; I-K; G, M). [*p-value <0.05; **p-value <0.001].

To further explore if a specific pattern of TFBS may be required for driving skeletal muscle specific expression, we mapped all TFBS conserved from mouse to human onto the 20 predicted SMEs. Using the cluster analysis function of the MultiTF tool (http://multitf.dcode.org/), we found that 9/20 SMEs included clusters with at least one Mef2 and one MyoD sites within <200 bp regions. All 6 SMEs validated had these features as well as 1/3 of the *Krt8*-only validated elements (SME11). Of all predicted SMEs, two elements that scored negative in the transgenic assay, (SME6/20) had this feature (Fig. 4). To functionally link the Mef2 and MyoD target sites to muscle-specific expression, we created two new constructs that included both the kidney enhancer and the muscle enhancers SME8 or SME16 within the Tol2*-γ-cry* vector (Fig. 5A). SME8 had a cluster of 1 Mef2 and 2 MyoD within 100 bp, and one additional Mef2 site >100 bp away (Fig. 5B). SME16 had only one cluster of a Mef2 and a MyoD predicted site present within a 100 bp DNA fragment (Fig. 5H). Next, we systematically deleted the Mef2, the MyoD or both the Mef2/MyoD sites present within the 100 bp region in SME8 (Fig. 5C–F) and SME16 (Fig. 5I–L) and examined transgenic frequency and expression intensity in both kidney and skeletal muscle (Table S3).

Enhancer proximity to the promoter influenced the ‘tissue-specificity’ activation level, such that the largest number of transgenic embryos expressing unaltered SME8 or SME16 constructs had muscle expression: 100% for SME8 (73% muscle only; 27% muscle and kidney; 0% kidney only) and 74% for SME16 (26% muscle only; 38% muscle and kidney; 5% kidney only). All deletion constructs had a decrease in the % of transgenic animals that expressed in the muscle, as well as a decrease in the expression intensity (Fig. 5G). As the muscle enhancer activity ‘weakened’ due to TFBS deletions, the kidney enhancer activity increased. When either the Mef2 or MyoD sites were deleted in SME8 the percentage of transgenic embryos expressing exclusively in skeletal muscle tissue decreased by ∼45% and the percentage of transgenic expressing in the kidney increased by ∼50% (Fig. 5D–E). Deleting the 73 bp region containing a cluster of 1 Mef2 and 2 MyoD sites in SME8 dramatically reduced the number and intensity of the muscle expression to 18% and the kidney expression increased to 39% (Fig. 5F). The remaining embryos that expressed in the muscle, had an average expression intensity 85% below the intensity observed in the intact SME8 transgenic embryos (Fig. 5G).

For SME16, deleting the Mef2 site reduced the number of muscle expressing embryos by 97% and increased the number of kidney expressing embryos 7-fold (Fig. 5J). Deleting MyoD had a less dramatic effect on the overall % of transgenic embryos expressing in the muscle, and the expression intensity was unaffected (Fig. 5K, M). Deleting both the Mef2 and MyoD sites in SME16 reduced the muscle transgenics to 13%, and the muscle expression was half of that detected for the unaltered SME16 construct (Fig. 5L, M). In addition to observing a reduction in the transgenic frequency, all deletion constructs but SME16_ΔMyoD had significantly less intense muscle expression relative to the wildtype constructs, suggesting that these sites are required for robust transgene activation. As the muscle expression dimmed, the kidney expression increased, suggesting that the enhancers are slightly competing with each other and that the enhancer closer to the promoter is favored. While enhancer competition has not yet been thoroughly investigated in vertebrates, it has been documented for many loci in Drosophila [36], and promoter competition has been observed in humans [37], [38]. However, the enhancer blocking activity of the proximal enhancer diminished with loss in activation potency due to Mef2 and MyoD mutations.

## Discussion

In this study we aimed to develop a high throughput transgenic system for validating tissue-specific enhancer function *in vivo*, in real time, in a vertebrate organism, and overcome some of the limitations posed by current technologies in the mouse and zebrafish model organisms. The optimization focused on: transgenic efficiency, promoter sensitivity and specificity, as well as reporter stability and expression intensity to generate a universal transgenic construct that is silent or ‘off’ in the absence of a tissue specific regulatory element, yet is capable of activating the reporter gene in a highly specific manner even when combined with a ‘weak’ tissue-specific enhancer. Initially we focused on transgenic methods in *Xenopus* using viability and transgenic efficiency as a metric. Side by side comparisons of nuclear gene transfer (REMI) and two transposon-mediated transgenic systems: PiggyBAC and Tol2 found both transposable element systems to be superior in embryo viability relative to REMI; however Tol2 yielded the largest percentage of transgenic embryos among the 3 groups (Fig. 1B). Among reporter genes, we chose to examine a family of red fluorescent proteins that are near the infrared range, to identify the brightest, most stable with least associated auto-fluorescence in *Xenopus*. We found Katushka and subsequently the monomer mKate2 to be the brightest and with the least non-specific signal generated by the *Xenopus* tissue (Fig. 1A).

Using a previously described enhancer prediction method we evaluated whether: 1) this *in silico* approach can reliably distinguish enhancers active during vertebrate skeletal muscle development and differentiation and 2) whether *Xenopus* transgenesis can be efficiently employed to validate tissue specific enhancers. Among the 20 tested elements from the 190 predictions across the mouse genome, 9 showed muscle-specific activity either in combination with *γ*-crystalin, keratin-8 or both promoters, 2 predicted enhancers exhibited expression patterns in a tissue other than the predicted site (skin and brain), and the remainder 9 elements did not have a detectable enhancer activity in transgenic *Xenopus*, examined up to stage 45. These results suggest that *in vivo* validation of muscle-specific enhancer predictions in transgenic frogs had a success rate of 45%. Furthermore, it is important to note that the computation approach employed represents a great improvement upon using evolutionarily conservation as the only selection criteria for candidate tissue-specific enhancers. Fig. 3A–F depicts the human/mouse conservation profile for the genetic loci corresponding to the neighboring gene of the validated SMEs. Each locus has a minimum of 3 conserved noncoding sequences and as many as 23 such elements, therefore the enhancer identification method significantly enriched for elements with tissue-specific activity and represents an important prioritization tool for determining which elements should be validated *in vivo*, in costly, laborious functional assays.

More recently there have been new computational classifiers that can further improve the predictive power of tissue specific enhancers [39]. One recent report used a training set of validated cardiac enhancers to predict cardiac enhancers *a priori* across the human genome, and cross-validation in Zebrafish demonstrated that 62% of the predictions corresponded to heart enhancers [39]. A future goal of our work is to apply these 9 newly described skeletal muscle enhancers along with others described in the literature to refine skeletal muscle predictors and improve reliability. One important distinction is worth noting is the homogeneity of expression we observed among the validated enhancers in *Xenopus*, using the Tol2-construct we engineered. In general, most previous reports of validated tissue-specific enhancer predictions in zebrafish or mouse transgenics, the predicted tissue is not generally the only positive tissue associated with the confirmed enhancers, nor is the pattern of expression always the same. For example, among the cardiac enhancers that were validated in mice [39], some enhancers gave rise to distinct spatial domains of expression outside the heart field. This spatial heterogeneity could be the result of several factors: 1) position effect where the transgene is influenced by the genomic context of the site of DNA integration; 2) promoter permissiveness where certain ‘minimal promoters’ are prone to activation bias, i.e. they sporadically turn on in a certain cell lineages, or 3) the modular nature of transcriptional regulatory elements, each of which corresponds to a different array of transcription factor binding sites that cumulatively contribute to the spatial specificity of an element. What the computation method recognizes as a tissue specific enhancer is a combination of TFBSs that together correspond to a shared character of the DNA elements examined. However, the predicted elements may include other unrecognized features that also contribute to the modular function of these elements. Nonetheless, it is worth highlighting that 100% of the skeletal muscle enhancers validated in the Xenopus transgenic strategy described here had an indistinguishable expression pattern, namely in the fast muscle fibers that are oriented in highly organized chevrons along the notochord in the *Xenopus laevis* tadpole. Also, most of the expression was in the trunk region and up to somite ∼25, suggesting that the expression of these regulatory elements marks a subset of muscle cells which does not extend to the slow muscle fibers that develop in the posterior-to-anterior direction and emerge at the 30 somite stage [40]. Such high cellular specificity in enhancer activity can now be further explored to determine the underlining sequence signatures responsible for this activity. Future studies using training sets that correspond to highly specialized subsets of enhancers will likely uncover a different collection of predicted enhancers, with distinct spatial specificities in a given tissue.

Earlier work by Wasserman and Fickett employed linear and logistic regression to computationally predict DNA regions specific to genes expressed in muscle [41] based on the presence of position weight matrices defined for a set of muscle-specific transcription factors including: Mef2, Myf, Sp1, SRF, and Tef transcription factors. Whereas these studies predicted regions with cohorts of these muscle-specific TFBS in the promoters of genes known to be expressed in muscle, the predicted elements were correlative, and were not functionally validated to confirm first the enhancer activity and second the contribution of individual TFBS to the enhancer activity and tissue specificity. The *Xenopus* transgenic assay however, can now facilitate the systematic mutagenesis of these sequence signatures to determine which sites are essential or additive in nature. Using the principle that 1 or more of these TFBS contribute to the ‘strength’ and ‘tissue-specificity’ of these elements, we deleted MyoD and Mef2 TFBS from two of the validated enhancers and showed that in both cases removing the individual TFBS or the whole combinatorial module diminished the enhancer activity by decreasing expression intensity and tissue-specific transgenic efficiency.

This study identified novel skeletal muscle enhancers likely to play critical roles during myogenic differentiation and muscle physiology. Our computational predictions of mammalian enhancers that have potential tissue specific activity combined with the transgenic tools we developed in *Xenopus* are likely to facilitate the validation and characterization of tissue-specific elements. The *in vivo* validation represents a new and cost effective *in vivo* enhancer screening tool which can help elucidate the mechanisms of gene regulation with the ultimate goal of determining the genetic variation that contributes to normal physiology and disease.

## Materials and Methods

### Transgenesis

Restriction enzyme mediated transgenesis by sperm nuclear transfer was performed as previously described [19], [42]. Briefly, 4 µL of gel purified insert DNA (150–250 ng/µL) was mixed at room temperature (RT) with 5 µL sperm nuclei (1.25×10^8^ nuclei/mL), incubated for 5 min and diluted with 23 µL of sperm dilution buffer, 4 µL MgCl_2_ (50 mM) and 0.5 µL *Not*I restriction enzyme, after a 10 min incubation at RT the mixture was transferred to a chilled aluminum block. De-jellied fresh eggs were washed 5x with chilled 1X MMR, transferred to 0.2X MMR +4% Ficoll in agarose injection dishes, and injected with the sperm nuclei suspension. Injected embryos were transferred to new dishes with fresh 0.2X MMR +4% Ficoll +100 µg/mL Gentamycin and were incubated at 15°C for 12–16 hours, after which healthy embryos are transferred to 0.2X MMR +100 µg/mL Gentamycin and monitored for gene expression using fluorescence. PiggyBAC transgenics were generated as previously described [14]. Tol2 tranposase RNA was prepared using *in vitro* transcription of Xba I-digested pT3TS/Tol2 plasmid (a gift from Dr. S.Ekker) [43] using T3 Ambion mMessage Machine kit. Transgene DNA was prepared using Qiagen midi-prep kits and stored in RNase-free water at 4°C. RNA and DNA were mixed 1∶1 and 125–150 pg of DNA and RNA each were injected in a 3 nL volume using a PicoSpritzer III (Parker Instrumentation). Embryos were injected near the sperm entry point in 2.5% ficoll in 1/3 MR at 12°C. Post injection the embryos are transferred to agarose coated dishes containing 1/3 MR +50 µg/mL Gentamycin at 12°C up to 16 cell stage, then transferred to 15°C-RT and scored for fluorescence. Tadpoles were scored and photographed between stages 38 and 40. Each tadpole was examined for expression sites on both sides. Photos were taken on a Leica MZ16FA fluorescence dissecting scope using Image Pro Plus software, and bright field and fluorescent images were superimposed using either GIMP or Adobe PhotoShop. Because images were not pseudocolored using grayscale intensity mapping, some autofluorescence was observed in the gut of overexposed embryos, particularly for constructs that had weaker expression like the *γ-cry* promoter only construct (Fig. 2D).

### Reporter Genes

Katushka or TurboFP635 (Evrogen Cat No. FP721) is a 26 KDa dimeric far-red protein from sea anemone Entacmaea quadricolor with fast maturity and high photostability [13]. mKate2 (Evrogen Cat. No. FP181) is a similar far-red protein with a few slight differences: it is a monomer and it is documented to be ∼3X brighter than Katushka [44].

### Quantifying Fluorescence

Quantification of skeletal muscle transgene expression was performed using NIH ImageJ. The skeletal muscle region along the back and tail of the transgenic tadpoles was analyzed for mean red fluorescent intensity. Measurements were performed on four tadpoles for each transgene then normalized to the average intensity obtained from the un-mutated SME constructs. P-values were obtained using paired T-tests.

### Cloning

Ef1A promoter+GFP in the Tol2 pT2KXIG vector (gift from Dr. K. Kawakami) were replaced by CMV-Katuskha (Evrogen), and subsequently the CMV promoter was excised and replaced by *Krt8* (pKrt8eGFP-tol2; gift from Dr. V. Korzh), *γ-cry*, *β-globin* (β-globin-eGFP; gift from Dr. S. Ekker), *Hsp68* (pHsp68-LacZ; gift from Dr. D. Mortlock) and *Irx3* (pIrx-eGFP; gift from Dr. J. Skarmeta) promoters. KE was excised from pIrx-KE-eGFPVector (gift from Dr. J. Skarmeta), all predicted SMEs and the two positive control MEs were PCR amplified from mouse DNA and cloned in the EcoR1 site of pT2gK2 (JN418198) vector. All constructs were sequence verified.

### Accession Numbers

Vector-pT2gK2 (JN418198); SME2 (JN418199); SME3 (JN418200); SME4 (JN418201); SME8 (JN418202); SME12 (JN418203); SME14 (JN418204); SME16 (JN418205); SME18 (JN418206); KE (JN418207); SME8-ΔMef2 (JN613839); SME8-Δ2XMyoD (JN613840); SME8-Δ147-220 (JN613841); SME16-ΔMef2 (JN613842); SME16-ΔMyoD (JN613843); SME16-Δ220-290 (JN613844).

## Supporting Information

Figure S1 SME1 (A), SME10 (B), SME11 (C) showed muscle expression in combination with *Krt8* but not γ-cry promoter (yellow arrow). SME12 showed highly reproducible transgenic expression in the hindbrain (D; green arrow), and SME 14 in the skin (E; green arrow).
(TIF)

Table S1 Comparing survival and transgenic efficiency among 3 *Xenopus* transgenesis methods: nuclear transfer, PiggyBAC transposon mediate and Tol2 transposon mediated transgenesis. For each method 250 embryos were injected in 4 different experiments, and the number of surviving and transgenic embryos were recorded. A paired T-test was used to determine if there was a significant difference between the surviving and transgenics. For each method the expression intensity was evaluated in the transgenic embryos, and was classified as ‘ubiquitous’ (uniformly over the entire embryo), ‘low chimera’ (a small subset of the embryo had expression, or ‘high chimera’ (>50% of the embryo had expression).
(XLS)

Table S2 Muscle Enhancer Predictions generated by the EI method.
(XLSX)

Table S3 Tissue specific expression is compared among transgenic embryos carrying mutations in Mef2 and MyoD sites of SME8 and SME16 muscle enhancers. The number of embryos with specific expression patterns are indicated, the percentages are graphed in Figure 5.
(XLSX)
