# Supplementary figures and images for: Interrogating Transcriptional Regulatory Sequences in Tol2-Mediated Xenopus Transgenics
Source: PLoS One. 2013 Jul 16;8(7):e68548. doi: 10.1371/journal.pone.0068548 (PMC3713029; doi:10.1371/journal.pone.0068548)

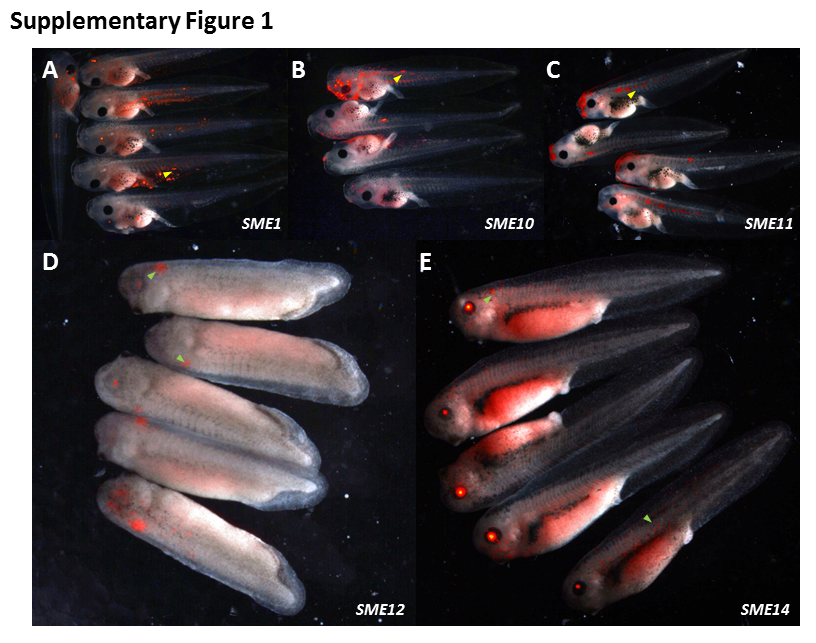

Supplement: Figure S1 — SME1 (A), SME10 (B), SME11 (C) showed muscle expression in combination with Krt8 but not γ-cry promoter (yellow arrow). SME12 showed highly reproducible transgenic expression in the hindbrain (D; green arrow), and SME 14 in the skin (E; green arrow). (TIF) [file pone.0068548.s001.tif]
